# Quantitative Assessment of ^99m^Tc-Depreotide Uptake in Oesophageal Cancer and Precursor Conditions and Its Reflection in Immunohistochemically Detected Somatostatin Receptors

**DOI:** 10.1155/2012/415616

**Published:** 2012-02-28

**Authors:** Gunnar Herlin, Lars Lundell, Åke Öst, Peter Aspelin, Leif Svensson, Rimma Axelsson

**Affiliations:** ^1^Division of Medical Imaging and Technology, Department of Clinical Science, Intervention and Technology, Karolinska Institutet, 141 86 Stockholm, Sweden; ^2^Department of Radiology, Karolinska University Hospital Huddinge, 141 86 Huddinge, Stockholm, Sweden; ^3^Gastrocentrum, Surgery, Karolinska University Hospital Huddinge, 141 86 Stockholm, Sweden; ^4^Division of Pathology, Karolinska University Hospital Huddinge, 141 86 Stockholm, Sweden; ^5^Division of Nuclear Medicine, Karolinska University Hospital Huddinge, 141 86 Stockholm, Sweden

## Abstract

*Background*. Somatostatin receptors (SSTRs) are over-expressed in several tumors making it possible for imaging with labelled SSTR. A previous study showed feasibility to image oesophageal cancer with SSTR analogue ^99m^Tc-depreotide. *Purpose*. (1) To investigate expression of the SSTRs in different types of esophageal carcinoma and (2) to correlate such an expression with ^99m^Tc-depreotide uptake in these lesions. *Material and Methods*. Total 28 patients (17 with esophageal cancer and 11 with Barrett's esophagus) were examined with ^99m^Tc-depreotide scintigraphy. The SSTR2A, SSTR2B, SSTR3, and SSTR5 were analyzed immunohistochemically in the lesion samples. *Results*. Among the patients with adenocarcinoma 10/11 expressed different amounts of SSTRs, while SSTRs were absent in 5/6 patients with Squamous cell carcinoma (Sqcc). There was no correlation neither between the ^99m^Tc-depreotide uptake and the amount of SSTRs nor between the amount of SSTRs and differentiation grade of the tumor. *Conclusions*. (1) SSTRs are expressed in esophageal carcinoma and more abundantly so in adenocancer specimens; (2) in vivo ^99m^Tc-depreotide uptake does not obviously correlate with the immunohistochemically detection of SSTRs of different subtypes in esophageal carcinoma.

## 1. Introduction

Cancer arises through a variety of mechanisms, and these different processes play an important role in tumour development and spread. By defining key pathways in those proliferative processes, the ambition has been to make it possible to target specific metabolic pathways or receptor steps, allowing tumour detection and collection of prognostic information relevant for diagnosis as well as treatment. Somatostatin receptors (SSTRs) occur in normal tissues like the brain, gastrointestinal channel, endocrine pancreas, kidneys, spleen, prostate, and thyroid. It is known that SSTRs can also be distinctly expressed in several tumours such as neuroendocrine tumours [1], tumours of the central nervous system, breast cancer, and lymphoid tissue [2, 3]. Somatostatin receptors are divided in six different subtypes: SSTR1, SSTR2A, SSTR2B, SSTR3, SSTR4, and 5 [4–6]. It is currently unclear to what degree and extent different receptor classes are expressed and overexpressed in the various neoplastic disease states. 

In this study, we investigated whether there was any correlation between the concentration of somatostatin receptors SSTR2A, SSTR2B, SSTR3, and SSTR5 in squamous cell carcinoma (SCC) and adenocarcinoma (Ac) compared to patients with Barrett's oesophagus without cancer. We investigated whether there was any correlation between these SSTRs and ^99m^Tc-depreotide uptake. We studied whether there was any correlation between these SSTRs and the differentiation grade of the tumour.

Depreotide is a somatostatin analogue binding to SSTR 2, 3, and 5 on cell surfaces. ^99m^Tc-depreotide scintigraphy has been shown to be a potentially valuable tool in the diagnosis of solitary pulmonary nodules and lymph node metastases [2, 7–10]. Our initial experience has also revealed that scintigraphic examination with ^99m^Tc-depreotide is feasible for imaging of oesophageal cancer [11].

The purposes of the present study were (1) to investigate whether the SSTRs are expressed in different types of oesophageal carcinoma as assessed by use of established immunohistochemical techniques; (2) to determine whether in vivo estimated ^99m^Tc-depreotide uptake correlates with the immunohistochemical detection of SSTRs of different subtypes in oesophageal carcinoma; (3) to explore whether there is a correlation between the expression of these SSTRs and the grading of the tumour; (4) finally, to address the question of whether the adenocarcinoma precursor condition, in the form of Barrett's oesophagus, contains these receptors, which can be displayed by ^99m^Tc-depreotide scintigraphy.

## 2. Material and Methods

### 2.1. Patients

Twenty-eight patients were enrolled in the study (7 females and 21 males with a median age of 63 years (range: 33–85 years)), Table 1. Among those, 17 had cancer of the oesophagus and 11 had Barrett's oesophagus (long segment, i.e., ≥3 columnar-lined oesophagus). The cancer diagnosis was established by histopathological examination of biopsy and/or operative specimens. All patients with Barrett's oesophagus were diagnosed with endoscopy and subsequent multiple biopsies.

 Among those 17 patients with cancer of the oesophagus, 11 had adenocarcinoma and 6 had squamous cell carcinoma. Six of the 11 patients with adenocarcinoma also had Barrett's oesophagus.

### 2.2. SSTR Scintigraphy

^99m^Tc-depreotide (740 MBq) was administered via an antecubital vein. Single-photon emission computed tomography (SPECT) of the thorax was performed at 2 h after injection, with the arms elevated, using three different gamma cameras. Most of the patients (20 of 28) were examined with a double-headed gamma camera (E-Cam, Siemens, Erlangen, Germany) and low-energy, high-resolution, and parallel-hole collimators, using a 128 × 128 matrix, 64 projections through 360° rotation, and an acquisition time of 40 s per projection. An additional 5 patients were examined with a double-headed gamma camera (DST-XL; Sopha Medical Vision Scandinavia AB, Gif-sur-Yvette, France) and low-energy, ultra-high-resolution, and parallel-hole collimators, using the same acquisition parameters as above. Finally, 3 patients were examined with a three-headed gamma camera (Picker IRIX, Cleveland, OH, USA) and low-energy, high-resolution, and parallel-hole collimators, using a 128 × 128 matrix, 60 projections through 360° rotations, and an acquisition time of 64 s per projection. Transverse slices were reconstructed with an iterative algorithm (HOSEM v 3.5 iterative programme; Hermes/NUD, Stockholm, Sweden) and formatted as a 128 × 128 matrix without attenuation correction. Images were postfiltered with a three-dimensional Fourier filter (Butterworth filter) with a cutoff frequency of 1.1 cycles/cm (order 5.00).

**Figure 1:**
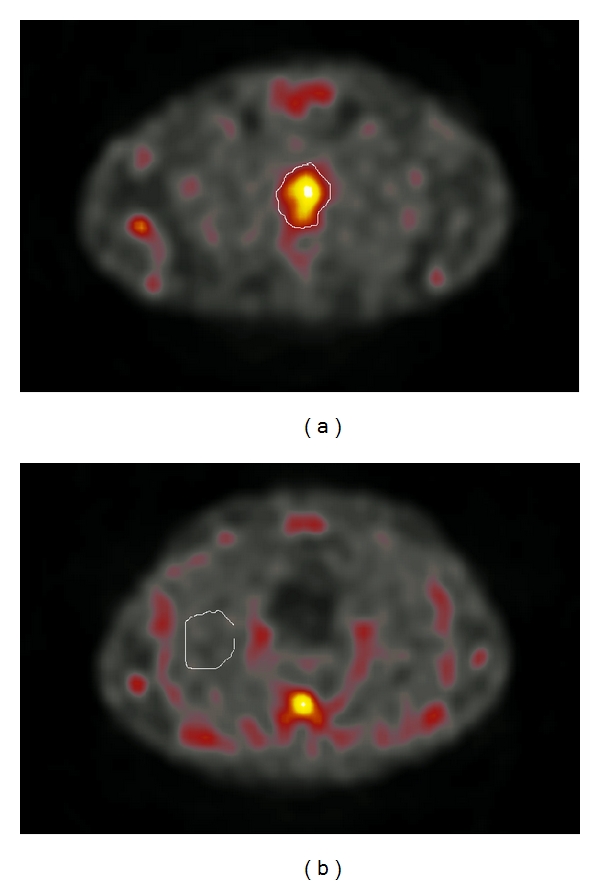
Evaluation of scintigraphic images with ^99m^Tc-depreotide. Region of interest (ROI) was drawn manually around the oesophageal tumour on each slice, using small margins, and a background ROI was drawn in healthy lung parenchyma.

The results were evaluated both through visual assessment and through quantitative calculations in the 2-hour images performed twice, in April 2009 and October 2009 by the same radiologist, who is a specialist in general radiology and experienced in nuclear medicine, and in November 2010 by a second radiologist, who is a specialist in both general radiology and nuclear medicine. CT scans were used for an accurate localisation of the ^99m^Tc-depreotide uptake and for placement of the region of interest (ROI). On visual assessment, any focal ^99m^Tc-depreotide uptake in the region of the known oesophageal lesion was considered pathological. The quantitative evaluation of ^99m^Tc-depreotide uptake was performed retrospectively on SPECT images in all 28 patients. First, an ROI was drawn manually around the oesophageal tumour on each slice, using small margins (Figure 1). Next, a background ROI was drawn in healthy lung parenchyma (Figure 1). A volume of interest (VOI) was obtained by adding all ROIs. Inhouse software, originally developed for volumetric measurements in magnetic resonance images and implemented on a Hermes workstation (Hermes Medical Solution AB, Stockholm, Sweden), was used to calculate the total counts and volume of the tumour and background VOIs, thus giving a count density [counts/cm^3^]. To produce a normalised tumour uptake, each patient was normalised to his or her own normal lung parenchyma using the formula *U* = (*T* − *B*)/*B*, where *U* is the normalised uptake, *T* is the count density in the tumour, and *B* is the count density in the lung parenchyma.

Both intraobserver and interobserver variability for the quantitative assessment of ^99m^Tc-depreotide uptake in the oesophageal lesions was low, with intraclass correlation coefficient (ICC) = 0.97 when comparing the evaluations by the same radiologist (intraobserver) and ICC = 0.96 when comparing the evaluations made by the two radiologists (interobservers).

### 2.3. Immunohistochemistry

For immunohistochemical assessment of the different SSTRs (2A, 2B, 3, and 5), the bond system (Vision Bio Systems Ltd Australian, Melbourne) was used. The antibodies were purchased from Gramsch Laboratories, Kirchenstraße 6, 85247 Schwabhausen, Germany.

Tissue specimens of the oesophageal tumours and biopsy material from the patients with Barrett's oesophagus were processed and prepared for immunostaining by use of monoclonal antibodies. The pretreatment to achieve the epitope was performed by heat treatment and with the enzyme pronase.

The tissue sample was first treated with peroxidase. The antibody was diluted 1000 times and the enzyme pronase was diluted 50 microlitres in 7000 microlitres. The tissue sample for SSTR2A and SSTR2B was pretreated with the diluted enzyme solution and with the enhancer for 10 min. The tissue sample for SSTR3 was pretreated with H1 = citrate buffer pH = 6, without enzyme and without enhancer for 20 min. and the tissue sample for SSTR5 was pretreated with H2 = EDTA buffer pH = 9 without enzyme and without enhancer for 40 min.

After this pretreatment the samples were incubated with the antibodies for 30 min. at a temperature between 37°C and 100°C. The development was then performed with diaminobenzidine (DAB) and then stained with haematoxylin.

The Bond Polymer Refine Detection System is a compact polymer system with high sensitivity, which includes peroxide block, intensive DAB dyeing, and haematoxylin contrast dyeing. This gave the dyeing high intensity combined with a sharp definition, without the use of streptavidin and biotin. This excluded the occurrence of nonspecific dyeing due to the presence of endogenous biotin, which occurs in large amounts in some tissues in the gastrointestinal channel. During testing of the antibodies, pancreas and skin were used as a positive control to exclude false positive results. Both positive and negative controls were used during the incubation and dyeing.

SSTR2A/SS800 was the antibody against SSTR2A, SSTR2B/SS860 was the antibody against SSTR2B, SSTR3/SS850 was the antibody against SSTR3, and SSTR5/SS890 was the antibody against SSTR5.

SSTR2A: SS800 from rabbit, COOH-terminus, titre 1 : 4000, specific for human, rat, and mouse; host: *rabbit*. ETQRTLLNGDLQTSI.
SSTR2B: SS860 from rabbit, COOH-terminus, titre 1 : 4000, specific for human; host: *rabbit.* FRNNKNRKK.
SSTR3: SS850 from rabbit, COOH-terminus, titre 1 : 4000, specific for human; host: *rabbit.* QERPPSRVA.
SSTR5: SS890 from rabbit, COOH-terminus, titre 1 : 4000, specific for human; host: *rabbit.* CQEAT RPRTA AANGL MQTSK L.

The enhancer was a copper intensification. The buffers used were H1 = citrate buffer, pH = 6, and H2 = EDTA buffer pH = 9.

The (SSTR) concentration was graded as no receptor presence = 0, that is, negative staining (grade 0). Small amounts = 1, that is, weak staining (grade 1) or only uneven (focally) positive. Moderate amounts = 2, that is, moderate staining or moderate positive (grade 2). Large amounts = 3, that is, strong positivity (grade 3).

**Scoring Procedure**

**Figure 2:**
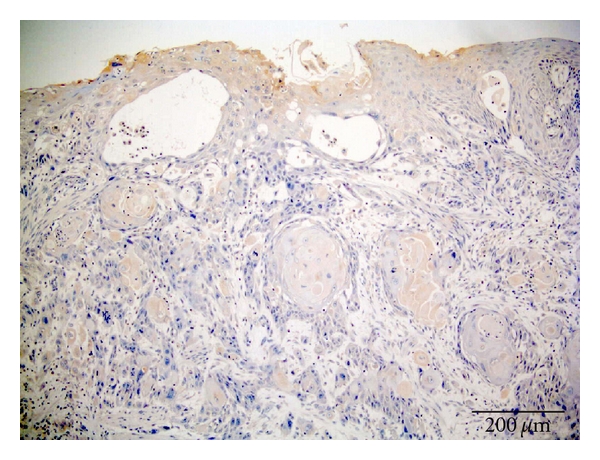
Staining SSTR3 SCC. Score 0.

**Figure 3:**
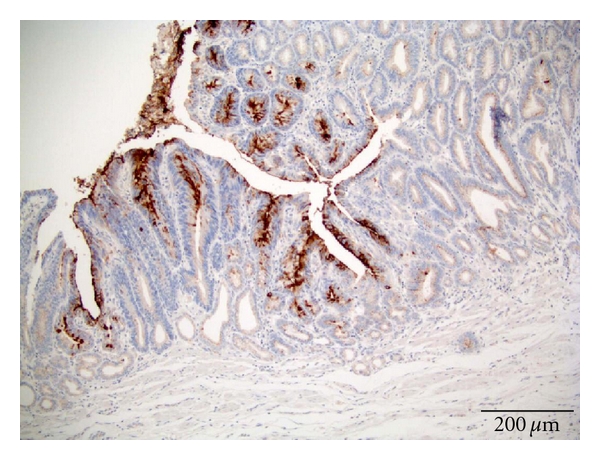
Staining SSTR3 adenocarcinoma. Score 1.

**Figure 4:**
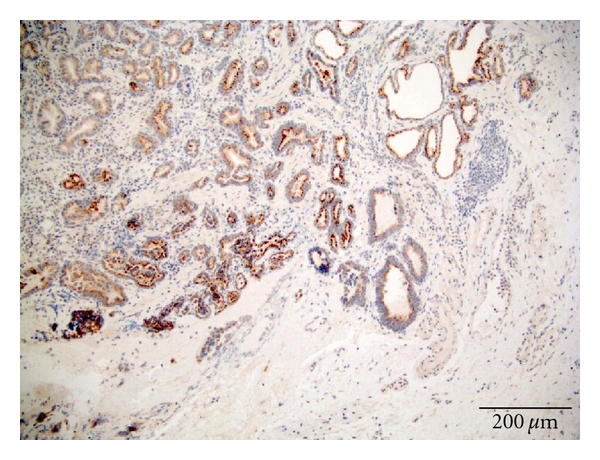
Staining SSTR3 adenocarcinoma. Score 2.

**Figure 5:**
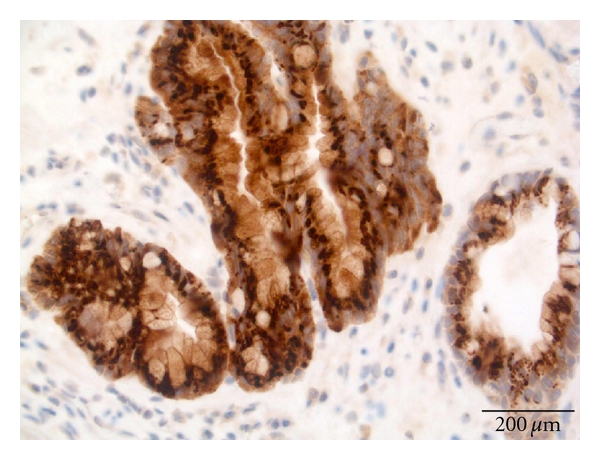
Staining SSTR3 adenocarcinoma. Score 3.

The stain scoring was made after comparison of a series of photos showing negative, slight, moderate, and strong staining results.Examples of those “standard photos” are shown in Figure 2, no receptor presence; Figure 3, small amounts; Figure 4, moderate amounts; Figure 5, large amounts.

### 2.4. Statistics

Because extreme values may bias results when only two variables are being examined, relationships between ^99m^Tc-depreotide uptake, tumour grade, and amount of the different studied SSTRs were analysed using Spearman rank correlations. Corresponding *ρ* values were calculated and considered significant if the *ρ* value was less than 0.05.

To assess intraobserver and interobserver variability, intraclass correlation coefficients were determined [12]. In the intraobserver variability, evaluations were performed twice, 6 months apart, by the same radiologist, and the mean value of the two uptake values was used in further analysis. In addition, a second radiologist made individual evaluations in order to assess the interobserver variability of the uptake values of the 2-hour images.

The study protocol was approved by the Regional Ethics Review Board in Stockholm, Sweden and the Radiation Safety Committee at Karolinska University Hospital, Huddinge.

## 3. Results 

One radiologist measured values for ^99m^Tc-depreotide uptake in April 2009 and October 2009, and a second radiologist measured these uptake values in November 2010. Both intraobserver and interobserver variability for the quantitative assessment of ^99m^Tc-depreotide uptake in the oesophageal lesions were low, with the ICC being 0.97 and 0.96, respectively.

Immunohistochemical detection and semiquantitative assessment of the different SSTRs and ^99m^Tc-depreotide uptake in 11 patients with adenocarcinoma are present in Table 2, those of the 6 patients with SCC are present in Table 3, and those of the 11 patients with Barrett's oesophagus without cancer in Table 4. The summary of these results is shown in Table 5. Among the 6 patients with SCC, only one patient displayed SSTR5, and the remaining 5 patients were devoid of SSTRs. Among the patients with adenocarcinoma, the majority expressed low amounts of SSTRs; one patient had none, a few had moderate amounts, and only one patient expressed high amount of SSTR5.

Concerning the relationship between SSTR expression and tumour grading, we were unable to reveal a correlation between the differentiation of the tumour and the expression of SSTRs, for either Ac or SCC. An exception was a significant (*ρ* ≤ 0.05) correlation (*r* = 0.70) between the presence of SSTR2B and the grading of the Ac; the higher amount of SSTR2B, the higher the grading of the tumour.

Overall, we observed significantly lower levels of SSTR2A SSTR2B, SSTR3, and SSTR5 in SCC compared to Ac (*ρ* = 0.001, *ρ* = 0.019,  *ρ* = 0.0002, *ρ* = 0.047, resp.).

The majority of the patients with Barrett's oesophagus expressed SSTRs in their columnar epithelium. The semiquantitative scoring on the abundance of SSTR did not reveal any separation of those epithelium with dysplastic morphological changes from those without.

We were unable to reveal any correlation between the ^99m^Tc-depreotide uptake and the expression of any of the examined SSTRs in the 17 patients with cancer of the oesophagus.

There was a tendency for low-differentiated tumours to have higher ^99m^Tc-depreotide uptake compared to highly differentiated Ac tumours, but this difference did not reach statistical significance.

SCC seemed to express lower ^99m^Tc-depreotide uptake compared to adenocarcinoma, but this difference could not be statistically substantiated.

Cases showing positive uptake with the scintigraphic method but negative results in the immunohistological analysis displayed no remarkable degree of inflammation on histopathological examination of the tissue specimens. Neither did we observe an SSRT immunostaining of the inflammatory cells present in the specimens, or even the noninflammatory cells (stroma cells, vessels, and others).

Among the patients with Barrett's oesophagus, 5 had either high or low grade of dysplastic changes in the columnar epithelium. There was a tendency towards higher ^99m^Tc-depreotide uptake in the epithelium with dysplasia than in that without dysplasia, but again, this difference could not be statistically verified.

## 4. Discussion 

Our previous studies in patients with nonsmall cell lung carcinoma (NSCLC) showing increased ^99m^Tc-depreotide uptake on scintigraphic images and immunohistochemically detected expression of SSTR2A [13] encouraged us to investigate patients with oesophageal carcinoma. The idea was based on similarity of cancer types, for example, SCC and identical localisation within the thoracic cavity. As our pilot study [11], we showed that it was feasible to image SCC and also Ac of the oesophagus with somatostatin receptor scintigraphy, utilising ^99m^Tc-depreotide. Thereafter, we continued to explore the tissue correlate to these in vivo observations by immunostaining of different somatostatin receptors in the respective tumours and even in a precancerous condition. One important prerequisite for the potential implementation of the scintigraphy technology was the high level of intra- as well as interobserver agreement in the assessments. However, coming back to the originally formulated issues, the following messages seem to be justified.

1. SSTRs are expressed in oesophageal carcinoma and more abundantly so in adenocarcinoma specimens.
2. In vivo ^99m^Tc-depreotide uptake does not obviously correlate with the immunohistochemical detection of SSTRs of different subtypes in oesophageal carcinoma.
3. There is a questionable and clinically irrelevant correlation between the expression of these SSTRs and the grading of adenocarcinoma.
4. Finally, we found that Barrett's columnar epithelium contains these receptors, which can be displayed by ^99m^Tc-depreotide scintigraphy.

Based on the fact that the columnar epithelium of the stomach harbours substantial amounts of somatostatin cells (D cells), it came as no surprise that we found SSTRs in adenocarcinomas and Barrett's oesophagus, but not in SCC. The variability among tumours and patients was unpredictable, and therefore, it can be assumed that our initial theory of introducing the idea that SSTRs are involved in key pathways for the development of these neoplastic processes cannot be supported by the present findings. The robustness and strength of these observations are reinforced by the fact that we carefully investigated many of the other subtypes of SSTRs, not previously determined for patients with NSCLC [13], such as SSTR2B, SSTR3, and SSTR5. Moreover, we were unable to find a clear correlation between the SSTR expression and the dysplasia scorings of the Barrett's cases. The present observation that in adenocarcinomas there might be an association between the grading of the tumour and the intensity of some of the somatostatin receptors to be stained can be either a finding obtained by chance or a logical result, based on the reasonable assumption that the more differentiated the tumour is, the more closely it resembles the “normal” columnar epithelium, where the D cells are quite abundant.

The expression of SSTRs of different subtypes in the presently investigated patients with oesophageal carcinoma did not correlate with the ^99m^Tc-depreotide uptake on the scintigraphic imaging. This is in accord with our previous study on patients with NSCLC [13]. Attempts have been made to explain and understand why tumours with high uptake of labelled somatostatin receptor analogue ^99m^Tc-depreotide in scintigraphic images do not regularly express SSTRs on immunohistochemical examination of relevant tissue specimens. Kwekkeboom et al. [14] and Machac et al. [15] suggested that the ^99m^Tc-depreotide uptake on scintigraphic images may be due to the presence of accompanying leucocytes or activated neuroendocrine cells around the tumour cells [14] or in the surrounding granulomatous tissue [15]. We tried to clarify this option by examining thoroughly the blocks from every one of our patients, concerning signs of inflammation and the content of inflammatory cells, stroma cells, and vessels. Doing that, we could not observe any deviation in a direction that could explain the lack of correlation between uptake and the SSTR density. What other explanations to these findings can be considered? Is it possible that ^99m^Tc-depreotide scintigraphy is more sensitive to detecting SSTRs than the corresponding immunohistochemical methods used? Does ^99m^Tc-depreotide bind nonspecifically to structures or receptors on the cell surface other than those residing in the D cells? Is expression of SSTRs a dynamic or stable process, and which of these are picked up by the scintigraphic technology? Many questions remain to be answered before this method could be implemented in clinical practice.

We noted a tendency towards low-differentiated tumours having higher ^99m^Tc-depreotide uptake, and this could be caused by nonspecific binding to areas of the cell surface, which could be more common on tumour cells with low differentiation compared to high differentiation. This corresponds to our previous observations in NSCLC [13], where poorly differentiated tumours had a higher ^99m^Tc-depreotide uptake. However, this tendency was not statistically significant, either [13]. In order to explore corresponding relationships in more detail, much larger study cohorts are required.

Concerning patients without cancer but with a precancerous condition, these were enrolled because it would be of special value to have a tool that could aid in the early detection of those who will subsequently develop neoplasia. Although we found somewhat higher ^99m^Tc-depreotide uptake in patients with dysplasia compared to those without, the overlap was substantial. Even in the immunohistochemical analysis, the tendency was there to show that those with dysplasia more often expressed SSTRs (16 of 17) compared to those without dysplasia (13 of 21). The clinical value of these findings has to be further explored and substantiated in larger patient samples and with longitudinal evaluation.

## Figures and Tables

**Table 1:** Histopathological and immunohistochemical analysis of 28 oesophageal lesions.

Pat no	Age	Gender	SSTR2A	SSTR2B	SSTR3	SSTR5	Diff	SCC	Ac	B
1	68	F	0	0	0	0	2	X		
2	63	F	1	1	1	1	2		X	
3	64	M	0	0	0	0	2	X		
4	75	F	0	0	0	0	1		X	X
5	70	M	1	0	1	1	1		X	
6	67	M	1	1	1	1	3		X	X
7	60	M	1	1	3	1	—	—	—	X
8	57	M	1	1	3	—	—	—	—	X
9	58	M	0	0	0	0	3	X		
10	61	M	0	0	0	0	1	X		
11	61	M	0	0	1	1	1		X	
12	67	M	1	0	1	1	1		X	
13	62	M	1	1	1	1	1		X	X
14	85	F	1	1	1	1	3		X	
15	58	M	1	1	1	3	1		X	X
16	60	F	1	2	2	2	3		X	X
17	33	M	2	2	2	1	2		X	X
18	48	M	0	0	0	0	1	X		
19	67	M	—	0	0	2	2	X		
20	78	M	1	1	2	3	—	—	—	X
21	70	F	1	—	1	—	—	—	—	X
22	73	M	1	2	1	2	—	—	—	X
23	60	M	0	0	0	1	—	—	—	X
24	48	M	—	0	0	—	—	—	—	X
25	62	M	1	1	1	2	—	—	—	X
26	66	M	0	0	0	—	—	—	—	X
27	73	M	1	0	1	1	—	—	—	X
28	65	F	2	2	2	2	—	—	—	X

F: female, M: male, Diff: differentiation grade of the tumour, 1: low differentiation grade, 2: moderate differentiation grade, 3: high differentiation grade, SCC: squamous cell carcinoma, and Ac: adenocarcinoma, B: Barrett's oesophagus. SSTRs were graded no presence = 0, small amounts = 1, moderate amounts = 2, and large amounts = 3.

**Table 2:** Depreotide uptake and immunohistochemical analyses of 11 adenocarcinomas of the oesophagus.

Pat no	SSTR2A	SSTR2B	SSTR3	SSTR5	Grade of differentiation	Barrett's	Depreotide uptake
2	1	1	1	1	Intermediate	—	235.84
4	0	0	0	0	Low	yes	208.29
5	1	0	1	1	Low	—	307.14
6	1	1	1	1	High	yes	8.52
11	0	0	1	1	Low	—	12.40
12	1	0	1	1	Low	—	173.17
13	1	1	1	1	Low	yes	160.64
14	1	1	1	1	High	—	58.41
15	1	1	1	3	Low	yes	109.19
16	1	2	2	2	High	yes	111.77
17	2	2	2	1	Intermediate	yes	12.31

**Table 3:** Depreotide uptake and immunohistochemical analyses of 6 SCCs of the oesophagus.

Pat no	SSTR2A	SSTR2B	SSTR3	SSTR5	Grade of differentiation	Barrett's	Depreotide uptake
1	0	0	0	0	2	—	40.38
3	0	0	0	0	2	—	137.38
9	0	0	0	0	3	—	62.84
10	0	0	0	0	1	—	107.99
18	0	0	0	0	1	—	37.02
19	—	0	0	2	2	—	82.13

SSTR expression was graded as none = 0, small amounts = 1, moderate amounts = 2, and large amounts = 3.

**Table 4:** Depreotide uptake and immunohistochemical analyses of 11 long segment Barrett's patients without cancer.

Pat no	SSTR2A	SSTR2B	SSTR3	SSTR5	Depreotide uptake
7	1	1	3	1	52.52
8	1	1	3	—	0
20	1	1	2	3	40.36
21	1	—	1	—	28.19
22	1	2	1	2	9.86
23	0	0	0	1	31.86
24	—	0	0	—	5.96
25	1	1	1	2	0.86
26	0	0	0	—	0
27	1	0	1	1	56.45
28	2	2	2	2	1.35

SSTR expression was graded as none = 0, small amounts = 1, moderate amounts = 2, and large amounts = 3.

**Table 5:** Summary of the ^99m^Tc-depreotide uptake and immunohistochemically determined SSTRs.

Diagnosis	SSTR2A average	SSTR2B average	SSTR3 average	SSTR5 average	Depreotide average uptake
Adenocarcinoma	0.9	0.8	1.1	1.2	127.1
Squamous cell carcinoma	0	0	0	0.3	78.0
Barrett's oesophagus	0.9	0.8	1.3	1.7	20.1
